# Hypermethylation in the promoter regions of flavonoid pathway genes is associated with skin color fading during ‘Daihong’ apple fruit development

**DOI:** 10.1093/hr/uhae031

**Published:** 2024-02-15

**Authors:** Jihua Xu, Lan Xiong, Jia-Long Yao, Peilei Zhao, Shenghui Jiang, Xiaohong Sun, Chaohua Dong, Hongyuan Jiang, Xinyue Xu, Yugang Zhang

**Affiliations:** College of Life Sciences, Key Laboratory of Plant Biotechnology of Shandong Province, Qingdao Agricultural University, Qingdao, 266109, China; College of Horticulture, Engineering Laboratory of Genetic Improvement of Horticultural Crops of Shandong Province, Qingdao Agricultural University, Qingdao, 266109, China; College of Horticulture, Engineering Laboratory of Genetic Improvement of Horticultural Crops of Shandong Province, Qingdao Agricultural University, Qingdao, 266109, China; The New Zealand Institute for Plant and Food Research Limited, Private Bag 92169, Auckland 1142, New Zealand; College of Horticulture, Engineering Laboratory of Genetic Improvement of Horticultural Crops of Shandong Province, Qingdao Agricultural University, Qingdao, 266109, China; College of Horticulture, Engineering Laboratory of Genetic Improvement of Horticultural Crops of Shandong Province, Qingdao Agricultural University, Qingdao, 266109, China; College of Life Sciences, Key Laboratory of Plant Biotechnology of Shandong Province, Qingdao Agricultural University, Qingdao, 266109, China; College of Horticulture, Engineering Laboratory of Genetic Improvement of Horticultural Crops of Shandong Province, Qingdao Agricultural University, Qingdao, 266109, China; College of Life Sciences, Key Laboratory of Plant Biotechnology of Shandong Province, Qingdao Agricultural University, Qingdao, 266109, China; College of Horticulture, Engineering Laboratory of Genetic Improvement of Horticultural Crops of Shandong Province, Qingdao Agricultural University, Qingdao, 266109, China; College of Life Sciences, Key Laboratory of Plant Biotechnology of Shandong Province, Qingdao Agricultural University, Qingdao, 266109, China; College of Life Sciences, Key Laboratory of Plant Biotechnology of Shandong Province, Qingdao Agricultural University, Qingdao, 266109, China; College of Horticulture, Engineering Laboratory of Genetic Improvement of Horticultural Crops of Shandong Province, Qingdao Agricultural University, Qingdao, 266109, China; Academy of Dongying Efficient Agricultural Technology and Industry on Saline and Alkaline Land in Collaboration with Qingdao Agricultural University, Dongying, 257300, China

## Abstract

Apple fruit skin color fading is not well understood although the molecular mechanism of skin color formation is well known. The red-fleshed apple cultivar ‘Daihong’ (DH) exhibited fading skin color during fruit development despite having a heterozygous R6 allele but lacking Red-TE for red fruit skin. In this study, transcriptomic analysis revealed the expression level of *MdMYB10* increased with fruit development whereas reduced expression levels of *MdMYBPA1*, *MdCHS*, *MdANS*, *MdUFGT*, *MdLAR*, and *MdANR* were observed, consistent with decreased levels of chalcone, anthocyanin, catechin, epicatechin, and procyanidin B2. Whole-genome bisulfite sequencing (WGBS) indicated a global gain in cytosine methylation levels and increased methylation in 5′ and 3′ flanking regions of genes and transposable elements (TEs), and in TE bodies in all CG, CHG and CHH contexts, especially the mCHH context, during fruit development. The increased DNA methylation was attributed to reduced expression levels of DNA demethylase genes, including *MdDME1*, *MdROS1*, and *MdROS2*. Association analysis revealed a significant negative correlation between promoter methylation levels of *MdCHS*, *MdCHI*, *MdMYBPA1*, and their respective transcript levels, as well as a negative correlation between promoter methylation levels of *MdCHS*, *MdCHI*, *MdANR*, and *MdFLS*, and the content of chalcones, naringenin-7-glucoside, epicatechin, and quercetin. Treatment with the DNA demethylation agent 5-aza-2′-deoxycytidine verified the negative correlation between DNA methylation and gene expression within the flavonoid pathway. These findings suggest that hypermethylation in promoter regions of genes of the flavonoid biosynthesis pathway is associated with the reduction of gene expression and flavonoid content, and fruit skin color fading during DH apple development.

## Introduction

Flavonoids, a group of plant secondary metabolites, encompass chalcones, flavanols, flavonols, flavanones, proanthocyanidins (PAs), and anthocyanins, which are present in various fruits, vegetables, and flowers [1]. Anthocyanins accumulate in vacuoles, giving rise to the various colors observed in different tissues and organs of plants. The biosynthetic pathway of anthocyanin and other flavonoids has been extensively studied [2, 3]. The pathway commences with phenylalanine and is catalyzed by several enzymes [4]. Chalcone synthase (CHS) catalyzes a 4-coumaroyl-CoA with three malonyl-CoA units, forming chalcone. Chalcone is subsequently converted to naringenin by chalcone isomerase (CHI). Flavanone 3-hydroxylase (F3H) then converts naringenin into dihydrokaempferol, which is further converted to dihydroquercetin by flavonoid 3′-hydroxylase (F3′H). Dihydroflavonol 4-reductase (DFR) converts dihydroquercetin into quercetin, and subsequently flavonol synthase (FLS) transforms quercetin into leucoanthocyanidin. Leucoanthocyanidin serves as a common substrate for anthocyanin and PA biosynthesis. Anthocyanidin synthase (ANS) catalyzes the conversion of leucoanthocyanidin to anthocyanidin, followed by addition of a glucose to produce anthocyanin [3, 5]. Furthermore, anthocyanidin reductase (ANR) converts anthocyanin to epicatechin, while leucoanthocyanidin reductase (LAR) catalyzes leucoanthocyanidin to catechin. Epicatechin and catechin can polymerize to form PAs. Studies on apple have demonstrated a positive association between the expression levels of most structural genes and red pigment accumulation [6–8]. In contrast, in pear and grape only the enzyme UDP-glucoseflavonoid glucosyl transferase (UFGT) controls a crucial step within the anthocyanin biosynthetic pathway and pigment accumulation [9, 10].

Transcription factors also play a vital role in anthocyanin biosynthesis. The main transcription factors involved are MYB, bHLH, and WD40, which form the complex MBW to regulate the expression of related genes encoding the biosynthetic enzymes [11]. In apple, MdMYB1 and MdMYB10 are the two key anthocyanin-related MYB transcription factors, independently discovered by different research groups [12–14]. Later studies verified that *MdMYB1* and *MdMYB10* are alleles of the same gene on chromosome 9, with different numbers of tandem repeats in the promoter region [15–17]. Three specific variants in the *MdMYB10* promoter have been identified to enhance anthocyanin accumulation. An upstream minisatellite sequence called the R6 repeat has been found to activate *MdMYB10* expression in leaves, flowers, and fruits, leading to increased anthocyanin content [15]. The insertion of an LTR/Gypsy transposable element (Red-TE) in the *MdMYB10* promoter is associated with *MdMYB10* expression and anthocyanin levels in mature fruit skin [18]. Additionally, a terminal-repeat retrotransposons in miniature TE (TRIM-TE) insertion in *MdMYB10* promoter enhances anthocyanin levels specifically in flower petals [19].

Apart from MdMYB10, other transcription factors, such as MdMYB9, MdMYB11, MdMYB12, and a PA1-type factor called MdMYBPA1, are specifically involved in PA synthesis [20–23]. Both MdMYB9 and MdMYB12 can interact with MdbHLH to enhance the activity of MdLAR, an enzyme involved in PA biosynthesis. A recent study suggested that in red-fleshed apples MdMYB9 and MdMYB12 can bind to the promoter of *MdMYBPA1*, and MdMYBPA1 can interact with MdbHLH33 to bind to the promoters of *MdLAR* and *MdANR*, promoting PA accumulation [23]. Furthermore, under low-temperature conditions, MdMYBPA1 can bind to the promoter of *MdANS* and *MdUFGT* in conjunction with MdbHLH33, resulting in enhanced anthocyanin accumulation [23].

Following biosynthesis, anthocyanin is transported to the vacuole for storage by several types of transporters, including glutathione *S*-transferase (GST), ATP-binding cassette (ABC) transporters, and multidrug and toxic compound extrusion transporters (MATEs) [24, 25]. Alongside biosynthesis and transportation, studies have shown that polyphenol oxidase (PPO), peroxidase (POD), and intracellular laccase (LAC) are three common enzyme families participating in anthocyanin degradation during the ripening stage of fruits [26–28]. Among these enzymes, PPO is primarily located in plastids, while anthocyanin degradation occurs in vacuoles. Therefore, it is less likely that PPO is responsible for anthocyanin degradation in plants [29, 30].

Increasing evidence suggests that DNA methylation, an essential epigenetic modification, plays a role in fruit color changes [31–34]. Studies on climacteric fruits like tomatoes and non-climacteric fruits like strawberries have shown a global loss of methylation during fruit development [31, 33]. Conversely, in non-climacteric sweet oranges, a global gain of methylation was observed during the fruit ripening process, which was attributed to reduced expression levels of DNA demethylase genes [32]. However, the dynamics of global DNA methylation during the development of climacteric apple fruits have not been thoroughly characterized. Nonetheless, extensive research on cytosine methylation in the promoters of *MdMYB1* and the subsequent gene expression changes has been conducted, focusing on anthocyanin accumulation in mature apple skins [7, 35–38]. The main findings indicate that hypomethylation in the promoter region leads to the activation of *MdMYB1* expression, resulting in increased anthocyanin production in fruit skins. In addition to *MdMYB1*, in a ‘Fuji’ mutant, reduced methylation levels in *MdMYB90-like* promoter activated transcription of the gene and increased anthocyanin content [39]. However, color fading is observed in some varieties during fruit development. For instance, anthocyanin accumulation in ‘Red Bartlett’ pears gradually decreases throughout fruit development, and studies have mainly focused on understanding the underlying transcriptional changes [40]. Similarly, in certain white-fleshed apple cultivars like ‘Qinguan’ and ‘Golden Delicious’, the fruitlets initially exhibited red skin color but later transitioned to green, only to turn red again in the case of ‘Qinguan’ [41]. Nonetheless, it remains unclear whether and how changes in DNA methylation are involved in the color-fading process during fruit development.

In this research, we observed that ‘Daihong’ (DH) fruit skin color exhibited a vibrant red color at early development stages (S1 to S3); however, the accumulation of red pigments dramatically reduced from S4 to S7, fading to green appearance at fruit maturity. Of *MdMYB1-1*, *MdMYB1-2*, and *MdMYB1-3*, only the allele *MdMYB1-1* co-segregates with red fruit skin phenotype, corresponding to the presence of the Red-TE insertion. *MdMYB1-2* and *MdMYB1-3* are present in yellow- or green-skin cultivars such as ‘Orin’, ‘Indo’, ‘Golden Delicious’, and ‘Mutsu’, with a limited transcription level under natural light [13, 17, 35]. In our study, we first analyzed the *MdMYB1/10* genotype of DH; then, through analyzing the flavonoid composition and transcriptional and methylation profiles, we discovered a global hypermethylation and higher methylation levels in promoters during fruit development. This methylation pattern was potentially influenced by upregulated DNA demethylase genes. Moreover, these changes in DNA methylation may be correlated with the expression profiles of genes involved in the flavonoid pathway and the specific content of flavonoid compounds. Our findings provide insight into how DNA methylation impacts apple skin color changes during fruit development.

## Results

### *MdMYB1/10* genotype in DH

**Figure 1:**
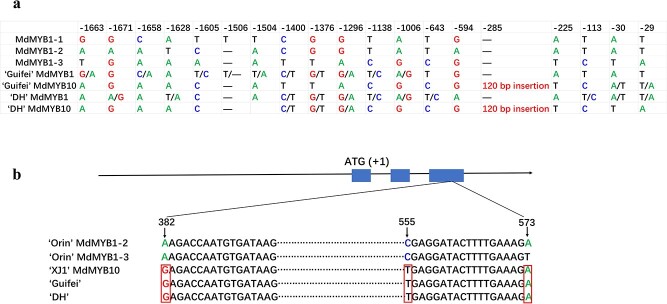
Polymorphisms of promoter and CDS sequences of *MdMYB1/10* in DH and ‘Guifei’. **a** Comparative analysis of polymorphisms of *MdMYB1/10* promoter. **b** SNPs in the third exon of the *MdMYB1/10* gene*.* ‘XJ1’ is a homozygous R6R6-type red-fleshed apple variety.

**Figure 2:**
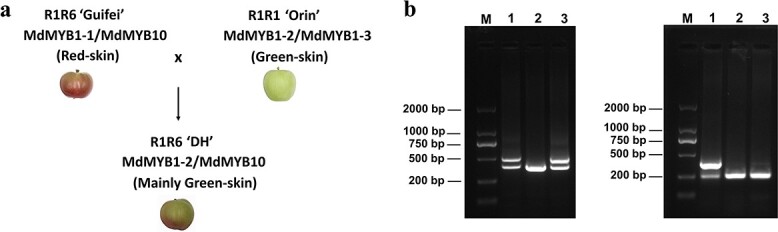
Analysis of promoter and upstream of *MdMYB1/10* in DH and its parents ‘Guifei’ and ‘Orin’. **a** Diagram summarized the alleles of *MdMYB1/10* in DH and its parents. **b** PCR analysis showed the presence and absence of R6 repeat (left panel) and Red-TE insertion (right panel) in the promoter of *MdMYB1/10* of ‘Guifei’ (1), ‘Orin’ (2), and DH (3). M, DNA marker.

The promoter region (~2000 bp from ATG) of *MdMYB1/10* from ‘Guifei’ and DH apple cultivars was PCR amplified, cloned, and sequenced. Sequence alignments of the promoter sequence with *MdMYB1-1* (DQ886414), *MdMYB1-2* (DQ886415), and *MdMYB1-3* (DQ886416) confirmed the presence of *MdMYB1-1* in ‘Guifei’ and *MdMYB1-2* in DH (Fig. 1). Notably, we observed a 120-bp insertion at position −285 bp in DH, containing five tandem repeats similar to the *MdMYB10* promoter reported previously [15]. These repeats, known as the repeat motif (R6), were inherited from the ‘Guifei’ parent (Figs 1a and 2a). PCR analysis confirmed the R6 repeat is heterozygously present in ‘Guifei’ and DH but absent in ‘Orin’ (Fig. 2b). Furthermore, we investigated the presence of the Red-TE insertion, known to be associated with red fruit color in apples [18]. PCR analysis using specific primers indicated the absence of the Red-TE insertion in DH and ‘Orin’, which is consistent with the absence of the *MdMYB1-1* allele in the two cultivars (Figs 1aand 2b). In contrast, the presence of the Red-TE insertion was observed in ‘Guifei’, corresponding to the existence of the *MdMYB1-1* allele in ‘Guifei’ (Figs 1a and 2b).

In addition, we cloned the cDNA of *MdMYB1/10* from ‘Guifei’ (R1R6), DH (R1R6), and a homozygous R6R6-type red-fleshed apple, ‘XJ1’ (R6R6), as well as the cDNA of *MdMYB1-2* and *MdMYB1-3* from ‘Orin’ (R1R1). Comparative analysis revealed that the cDNA sequence of *MdMYB10* in DH was identical to that of ‘Guifei’ and ‘XJ1’, but distinct from ‘Orin’ (Fig. 1b). This suggests that *MdMYB1-2* in DH is not transcribed.

### Fruit skin color and flavonoid contents during DH fruit development

**Figure 3:**
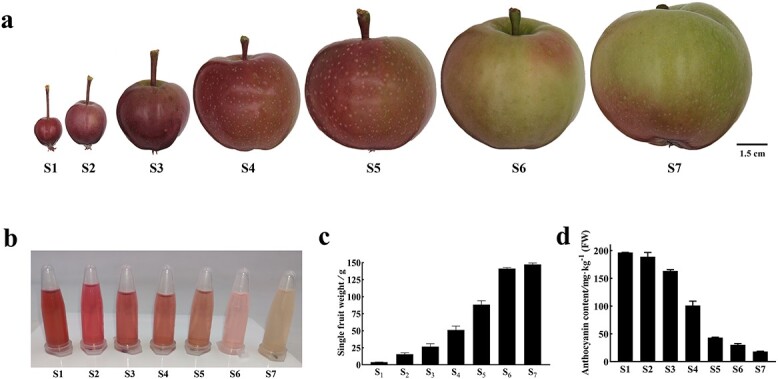
DH fruit phenotypic characters and skin anthocyanin content at seven developmental stages. **a** Fruit skin color fading at seven developmental stages. S1–S7 represent 30, 45, 55, 71, 90, 110, and 131 DAFB. **b**. Anthocyanin extracted from DH fruit skin. **c** Single fruit weight of DH. **d** Level of anthocyanin of DH fruit skin determined by the pH differential method. Error bars are standard deviations of three biological replicates.

The DH fruit skin color exhibited a vibrant red hue at stages S1, S2, and S3 (Fig. 3a and b). However, as fruit development progressed to S4 and S5, the accumulation of red pigments dramatically reduced, leading to a noticeable change in color towards greenish tones (Fig. 3a and b). At the mature stage (S7), the fruit weight reached its highest level, while the anthocyanin content was the lowest, contributing to the almost green appearance of the fruit skin (Fig. 3c and d).

Furthermore, the changes in flavonoid components during fruit development were investigated. S1, S4, and S7 stages were selected to represent the young, enlargement, and mature stages, respectively. In S1, in addition to enriched anthocyanin levels, higher amounts of flavanols, chalcones, flavanones, flavonols, and procyanidins were observed compared with S4 and S7 (Table 1). Remarkably, the content of catechin, a precursor for procyanidin formation, was nearly 10 times higher in S1 than in S7. Specifically, procyanidin B2 showed higher levels in S1 (494.19 mg/kg) compared with S7 (392.00 mg/kg). Phlorizin, a dihydrochalcone, exhibited the third-largest amount in S1 (477.35 mg/kg), which decreased with fruit development (Table 1).

**Table 1:** Analysis of flavonoids in DH fruit skin during fruit development.

**Compounds**	**Class**	**Molecular formula**	**Precursor ions [M-H]- or [M + H] + (*m*/*z*)**	**Theoretical mass (*m*/*z*)**	**S1** (**mg/kg)**	**S4** (**mg/kg)**	**S7** (**mg/kg)**
Hyperoside	Flavonols	C_21_H_20_O_12_	465.10	464.10	872.01	794.90	580.93
Procyanidin B2	Proanthocyanidin	C_30_H_26_O_12_	577.10	578.14	494.19	488.04	392.00
Phlorizin	Chalcones	C_21_H_24_O_10_	435.10	436.14	477.35	300.71	205.33
Quercitrin	Flavonols	C_21_H_20_O_11_	447.10	448.10	420.28	386.25	337.86
Avicularin	Flavonols	C_20_H_18_O_11_	433.10	434.08	196.76	172.72	134.30
Epicatechin	Flavanols	C_15_H_14_O_6_	289.10	290.08	102.19	111.66	80.93
Catechin	Flavanols	C_15_H_14_O_6_	289.10	290.08	88.40	16.41	9.18
Rutin	Flavonols	C_27_H_30_O_16_	609.10	610.15	81.87	76.52	51.91
Naringenin-7-*O*-glucoside	Flavanones	C_21_H_22_O_10_	433.10	434.12	33.49	12.06	5.30
Glycitin	Isoflavanones	C_22_H_22_O_10_	491.10	446.12	21.05	58.90	11.74
Sieboldin	Chalcones	C_21_H_24_O_11_	451.13	452.13	15.92	5.91	2.46
Quercimeritrin	Flavonols	C_21_H_20_O_12_	463.09	464.10	10.96	10.20	8.50
Afzelin	Flavonols	C_21_H_20_O_10_	431.10	432.11	7.35	3.99	2.46
Gallocatechin	Flavanols	C_15_H_14_O_7_	305.06	306.07	3.48	0.83	0.21
Taxifolin	Flavanonols	C_15_H_12_O_7_	303.10	304.06	3.36	0.00	0.00
Quercetin	Flavonols	C_15_H_10_O_7_	301.10	302.04	2.64	0.40	0.11
Eriodictyol	Flavanones	C_15_H_12_O_6_	287.10	288.06	2.57	0.23	0.17
Phloretin	Chalcones	C_15_H_14_O_5_	273.10	274.08	2.54	0.16	0.04
Total					2836.41	2439.90	1823.41

This result indicates that DH apple undergoes significant phenotypic changes and variations in flavonoid components during different stages of fruit development. The color transformation from red to green, accompanied by decreased anthocyanin content, signifies the maturation process. Additionally, the levels of flavanols, chalcones, flavanones, flavonols, and procyanidins show distinct fluctuations throughout fruit development.

### Changes in expression of flavonoid pathway genes during DH fruit development

**Figure 4:**
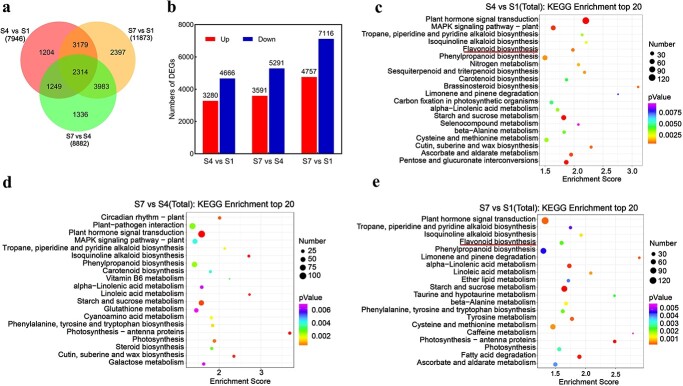
Transcriptome analysis of DH fruit skin at three developmental stages. **a** Venn diagrams of DEGs in three pairwise comparisons. **b** Numbers of upregulated and downregulated DEGs in three pairwise comparisons. **c, d, e** KEGG pathway enrichment analysis of DEGs in S4 versus S1 (**c**), S7 versus S4 (**d**), and S7 versus S1 (**e**). S1, S4 and S7 represent 30, 71 and 131 DAFB.

Transcriptome analysis revealed significant changes in gene expression patterns from S1 to S7. A total of 11 873 differentially expressed genes (DEGs) were detected in the comparison between S7 and S1, the highest number of DEGs among the three pairwise comparisons (Fig. 4a). Interestingly, there were more downregulated genes than upregulated genes in all three comparisons (Fig. 4b). Kyoto Encyclopedia of Genes and Genomes (KEGG) analysis of DEGs revealed enrichment of flavonoid biosynthesis pathway genes in comparisons of S4 versus S1 and S7 versus S1, but not S7 versus S4 (Fig. 4c–e).

**Figure 5:**
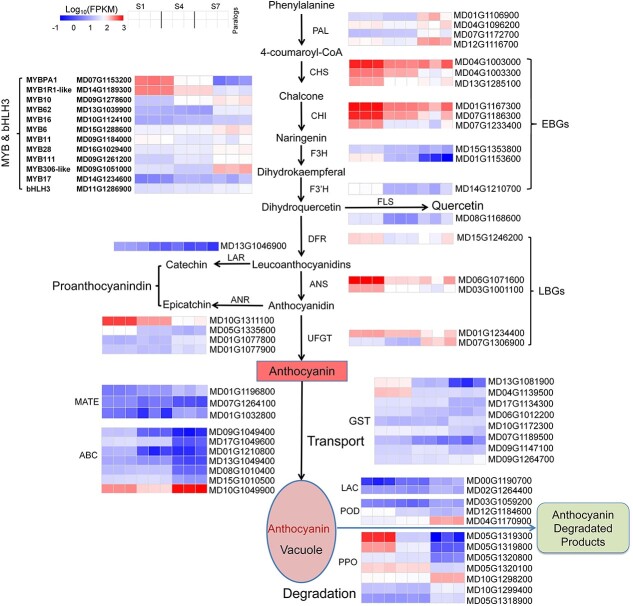
Expression profiles of DEGs involved in the flavonoid biosynthesis, transport, and degradation pathway. EBGs, early biosynthetic genes; LBGs, late biosynthetic genes. The color scale at top left ranging from blue to red represents low to high levels of gene expression (log_10_ FPKM). PAL, phenylalanine ammonia lyase; CHS, chalcone synthase; CHI, chalcone isomerase; F3H, flavanone 3′-hydroxylase; F3′H, flavonoid 3′-hydroxylase; DFR, dihydroflavonol 4-reductase; ANS, anthocyanidin synthase; UFGT, UDP-glucoseflavonoid glucosyl transferase; MATE, multidrug and toxic compound extrusion transporter; ABC, ATP-binding cassette; GST, glutathione *S*-transferase; LAC, laccase; POD, peroxidase; PPO, polyphenol oxidase.

Furthermore, we characterized the expression profiles of DEGs related to the flavonoid biosynthesis pathway (Fig. 5). Specifically, we focused on early biosynthetic genes (EBGs) and late biosynthetic genes (LBGs) in anthocyanin biosynthesis. The expression levels of EBGs, such as *MdCHS*, *MdCHI*, *MdF3H*, and *MdF3′H*, were found to significantly decrease with fruit development. Similarly, the expression of LBGs, including *MdDFR*, *MdANS*, and *MdUFGT* (MD01G1234400), showed significant downregulation, except for *MdUFGT* (MD07G1306900), which exhibited upregulation. We further investigated the expression patterns of genes participating in the biosynthesis of flavonols and PAs, and found *MdFLS*, *MdLAR*, and *MdANR* were significantly downregulated during fruit development.

Moreover, we examined the expression profiles of regulatory genes, particularly those encoding MYB transcription factors. The expression levels of several *MYB* genes were found to be dynamically regulated during fruit development, including reduced expression levels of *MdMYBPA1*, *MdMYB11*, and *MdMYB1R1-like*, but increased expression levels of *MdMYB6*, *MdMYB16*, *MdMYB17*, *MdMYB28*, *MdMYB111*, and *MdMYB306-like* (Fig. 5). Notably, the expression of *MdMYB10*, a positive regulator, showed an interesting pattern. It was upregulated during fruit development and reached its highest expression level at the mature stage, when anthocyanin content was the lowest.

Additionally, genes associated with anthocyanin transport, such as *MdGST* and *MdABC*, showed downregulation during fruit development, indicating a decreased capacity for anthocyanin transportation. On the other hand, genes involved in anthocyanin degradation, including *MdLAC*, *MdPOD*, and *MdPPO*, exhibited upregulation, suggesting enhanced degradation processes at the mature stage (Fig. 5). The downregulation of the transport genes and upregulation of degradation genes might also contribute to the reduced level of anthocyanin accumulation at the mature fruit stage.

To determine the validity of gene expression data obtained from RNA-seq, 19 of the DEGs participating in flavonoid biosynthesis, transport, and degradation were selected for qRT–PCR analysis (Supplementary Data Fig. S1). The results showed consistent expression patterns from S1 to S7 between the data generated by RNA-seq and qRT–PCR. This consistency validates the reliability of the transcriptome data. The relative expression levels of eight MYB transcription factors, including *MdMYB10*, determined by qRT–PCR were consistent with their transcript levels obtained by RNA-seq (Supplementary Data Fig. S1).

### DNA methylation profiles during DH fruit development

**Figure 6:**
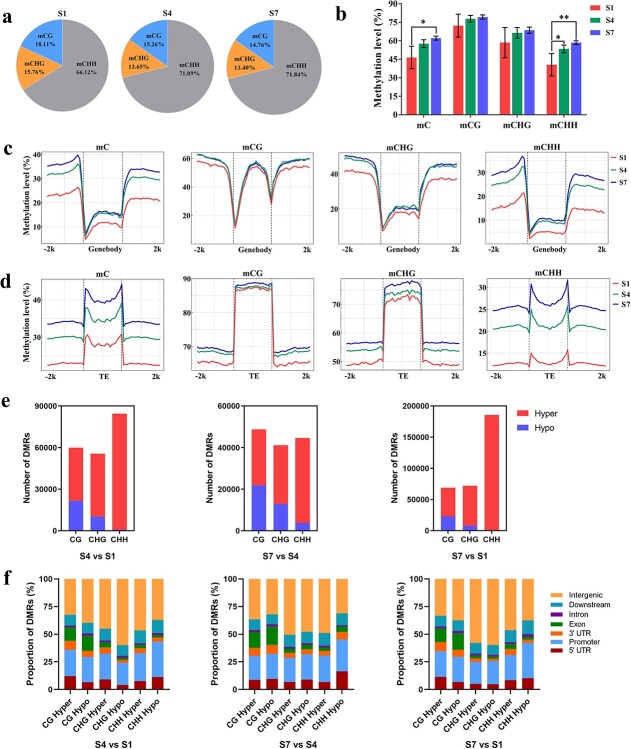
DNA methylation analysis of DH fruit skin tissues. **a** Relative proportions of mC, mCG, mCHG, and mCHH at development stages S1, S4, and S7. **b** DNA methylation levels of CG, CHG, and CHH at S1, S4, and S7. **c** DNA methylation levels of genes and their 2-K upstream and downstream regions at S1, S4, and S7. **d** DNA methylation levels of TEs and their 2-K upstream and downstream regions at S1, S4, and S7. **e** Number of hyper- and hypo-DMRs in S4 versus S1, S7 versus S4 and S7 versus S1. **f** Proportion of DMRs in different gene features in S4 versus S1, S7 versus S4, and S7 versus S1. Error bars are standard deviations of three biological replicates. ^*^P < 0.05; ^**^P < 0.01.

To investigate DNA methylation profiles during DH fruit development, whole-genome bisulfite sequencing (WGBS) was performed at the three developmental stages S1, S4, and S7. The sequencing generated >171.75 million clean reads with 25.34 Gb from each sample, with at least 67.13% of reads mapped to a reference genome (Supplementary Data Table S1). Further analysis revealed distinct patterns of DNA methylation dynamics during apple fruit development. The relative methylation levels of both CG and CHG sequence contexts decreased, while CHH methylation levels increased with fruit development (Fig. 6a). Overall, the level of methylated cytosines increased during development, primarily driven by CHH methylation (Fig. 6b, Supplementary Data Fig. S2).

Furthermore, DNA methylation in the 5′ and 3′ regions (upstream or downstream 2 K) of genes and TEs and in the TE body gradually increased from S1 to S7 in all CG, CHG, and CHH methylation contexts, particularly in the CHH methylation context (Fig. 6c and d). All S4 versus S1, S7 versus S4, and S7 versus S1 comparisons revealed a higher number of hyper-differentially methylated regions (hyper-DMRs) compared with hypo-DMRs in all contexts of CG, CHG, and CHH. The ratio of hyper-DMRs to hypo-DMRs was the highest in S7 versus S1 (Fig. 6e), especially for the CHH context, which showed the lowest hypomethylation numbers in all three comparative groups (Supplementary Data Fig. S3). These findings were in line with the RNA-seq expression data, which showed a greater number of downregulated genes compared with upregulated genes in all S4 versus S1, S7 versus S4, and S7 versus S1 comparisons, with a higher ratio of downregulated genes in S7 versus S1 (Fig. 4b). Regarding the genomic distribution of DMRs, ~50% of DMRs were located in intergenic regions in all S4 versus S1, S7 versus S4, and S7 versus S1 comparisons, followed by the promoter region (Fig. 6f). Notably, the promoter region exhibited a preferential enrichment of CHH hypo-DMRs (Fig. 6f). The KEGG analysis of DMRs indicated the enrichment of flavonoid biosynthesis pathway in all S7 versus S1, S4 versus S1, and S7 versus S4 comparisons for CG, CHG, and CHH contexts (Supplementary Data Fig. S4).

Overall, the results provide novel insights into the DNA methylation dynamics during DH fruit development in climacteric apple, revealing context-specific changes and their association with relative expression patterns.

### DNA methylation profiles in promoter of genes involved in flavonoid biosynthesis during DH fruit development

**Figure 7:**
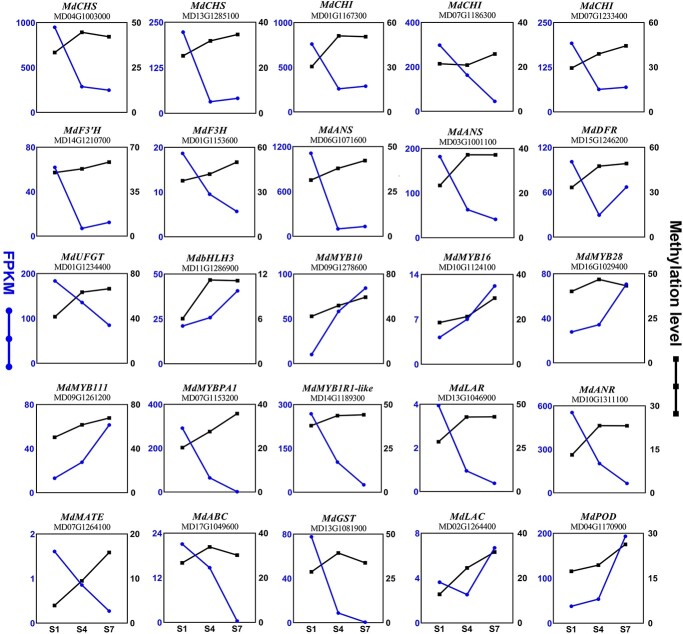
DNA methylation and expression levels of genes involved in anthocyanin and proanthocyanidin accumulation. Blue lines represent transcript levels (FPKM), and black lines represent methylation levels (%).

As DNA methylation in the promoter region can influence gene expression, we correlated the methylation levels with transcript levels of the main genes in PAs and anthocyanin biosynthesis and degradation. The structural genes *MdCHS*, *MdF3H*, *MdCHI*, *MdANS*, *MdUFGT*, *MdANR*, and *MdLAR* exhibited an increasing trend in DNA methylation levels within their promoter regions, while their transcript levels decreased from S1 to S7 (Fig. 7). On the other hand, the regulatory genes *MdMYB10*, *MdMYB16*, *MdMYB28*, *MdMYB111*, and *MdbHLH3* showed an increasing pattern in DNA methylation levels from S1 to S7, accompanied by upregulated gene expression. Similar changes were observed in the anthocyanin degradation genes *MdLAC* and *MdPOD*, which showed increasing methylation levels and transcript levels from S1 to S7. Interestingly, the methylation levels of *MdMYBPA1*, *MdMYB1R1-like*, and *MdMATE* increased from S1 to S7, contrary to their expression patterns. The IGV image of increased promoter methylation levels from S1 to S7 of keys genes in flavonoid biosynthesis is shown in Supplementary Data Fig. S5.

To validate the promoter methylation changes of the above genes obtained from WGBS data, we selected *MdMYB10* and performed an McrBC–PCR experiment. McrBC is a methylation-dependent endonuclease that cleaves methylated DNA. The results showed no visible differences in the bands of S1 samples, indicating low methylation levels of the MR3 fragments (Supplementary Data Fig. S6). Partial cleavage of the MR3 fragments was observed at the S4 stage, while severe cleavage was observed at the S7 stage, indicating high methylation levels at S7. These findings were consistent with the methylation levels obtained from the WGBS data.

Additionally, we analyzed the promoter methylation levels of genes involved in anthocyanin accumulation from S1 to S7 in the CG, CHG, and CHH sequence contexts. The results revealed that the elevated DNA methylation during fruit development was primarily contributed by the CHH context (Supplementary Data Table S2).

### Correlation between DNA methylation level and flavonoid content

Given the role of DNA methylation in modifying gene expression, understanding its impact on downstream product accumulation can provide insights into phenotypic variations; thus we examined the correlation between DNA methylation changes of key genes and the accumulation of anthocyanins, as well as specific flavonoid compounds.

We first assessed the correlation between promoter methylation levels of key genes and anthocyanin contents. Analysis of the data revealed that approximately half of the genes displayed a significantly negative relationship between promoter methylation levels and anthocyanin contents (Supplementary Data Table S3). Next, we investigated the impact of DNA methylation on specific flavonoid compound levels. We selected eight metabolites, comprising chalcones, epicatechin, catechin, procyanidin B2, naringenin-7-glucoside, quercimeritrin, quercetin, and quercitrin. Correspondingly, we analyzed the methylation levels of their upstream genes, namely *MdCHS*, *MdCHI*, *MdFLS*, *MdANR*, *MdLAR*, and *MdMYBPA1*.

The results revealed significantly negative correlations between the methylation levels of the *MdCHS* (MD13G1285100) promoter and both its transcript levels and chalcone contents (*r* = −0.736 and −0.706, *P* < 0.05) (Table 2). Similarly, the methylation level of the *MdCHI* (MD01G1167300) promoter exhibited a significantly negative correlation with its transcript levels and naringenin-7-glucoside content (*r* = −0.820 and −0.847, *P* < 0.01) (Table 2). Furthermore, the methylation level of the *MdANR* (MD05G1335600) promoter displayed significantly negative correlations with the levels of both epicatechin and procyanidin B2 (*r* = −0.718 and −0.805, *P* < 0.05) (Table 2). Similarly, the methylation level of the *MdFLS* (MD08G1168600) promoter displayed a significantly negative correlation with quercetin content (*r* = −0.775, *P* < 0.05) (Table 2).

**Table 2:** Correlation analysis of promoter DNA methylation levels of flavonoid biosynthesis genes with corresponding transcript levels and metabolite contents.

**Gene**	**Gene ID**	**FPKM**	**Chalcone content**	**Naringenin-7-glucoside content**	**Catechin content**	**Epicatechin content**	**Procyanidin B2 content**	**Quercimeritrin content**	**Quercetin content**	**Quercitrin content**
MdCHS	MD04G1003000	−0.708^*^	−0.457	NA	NA	NA	NA	NA	NA	NA
MdCHS	MD04G1003300	0.126	−0.077	NA	NA	NA	NA	NA	NA	NA
MdCHS	MD13G1285100	−0.736^*^	−0.706 ^*^	NA	NA	NA	NA	NA	NA	NA
MdCHI	MD01G1167300	−0.820 ^**^	NA	−0.847 ^**^	NA	NA	NA	NA	NA	NA
MdCHI	MD07G1186300	−0.53	NA	−0.32	NA	NA	NA	NA	NA	NA
MdCHI	MD07G1233400	−0.364	NA	−0.308	NA	NA	NA	NA	NA	NA
MdLAR	MD07G1153200	−0.625	NA	NA	−0.524	NA	0.217	NA	NA	NA
MdANR	MD10G1311100	−0.147	NA	NA	NA	0.186	0.321	NA	NA	NA
MdANR	MD05G1335600	−0.475	NA	NA	NA	−0.718 ^*^	−0.805 ^*^	NA	NA	NA
MdMYBPA1	MD07G1153200	−0.722 ^*^	NA	NA	−0.636	−0.554	−0.537	NA	NA	NA
MdFLS	MD08G1168600	−0.607	NA	NA	NA	NA	NA	−0.464	−0.775 ^*^	−0.591

^*^P < 0.05;

^**^P < 0.01. NA represents not applicable.

These findings suggest that the promoter region methylation levels of key genes in the flavonoid pathway are associated with the accumulation of specific flavonoid compounds. The negative correlations observed between DNA methylation levels and flavonoid contents indicate a potential regulatory role of DNA methylation in modulating flavonoid biosynthesis.

**Figure 8:**
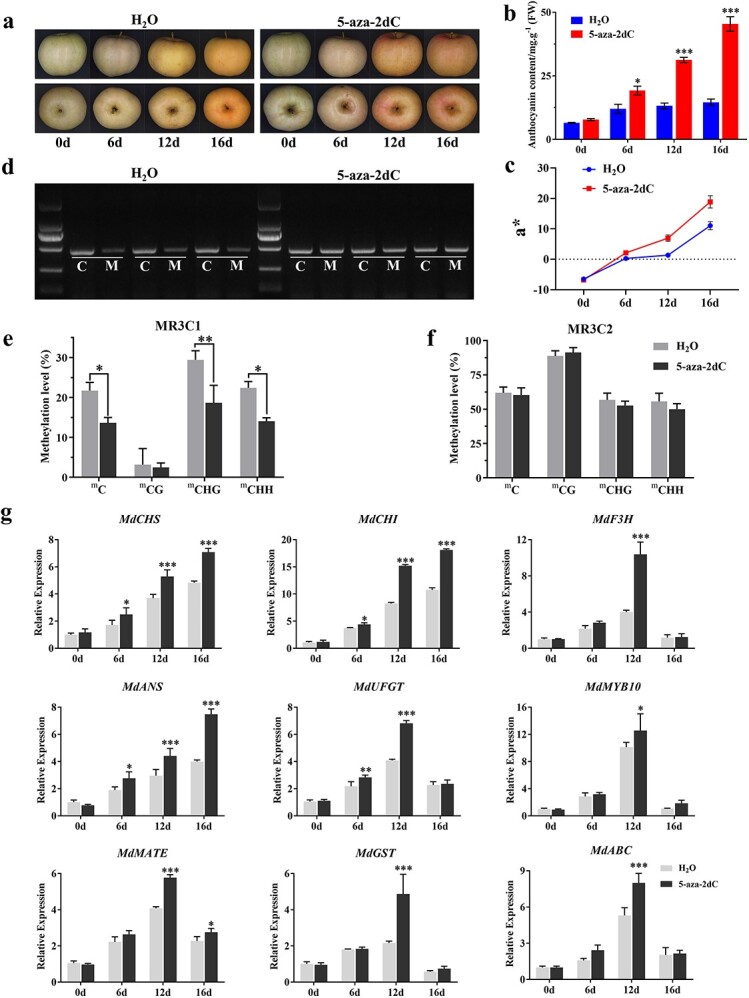
DNA methylation inhibitor treatment of DH apple fruit skin. **a** Color changes of DH fruit after treatment with water and the methylation inhibitor 5-aza-2dC. **b** Anthocyanin content of apple fruits after treatment with water and 5-aza-2dC. **c** The a* values of apple fruits were measured by using a colorimeter after treatment with water and 5-aza-2dC. **d** PCR was carried out with primers for amplifying the MR3 region of the MdMYB10 promoter and DNA extracted from fruit skin treated with water or 5-aza-2dC. The DNA was digested by McrBC enzyme with GTP (M) or without GTP as a control (C). **e**, **f** DNA methylation levels of MR3C1 and MR3C2. **g** Relative expression level of genes involved in flavonoid accumulation. ^*^P < 0.05; ^**^P < 0.01; ^***^P < 0.001.

**Figure 9:**
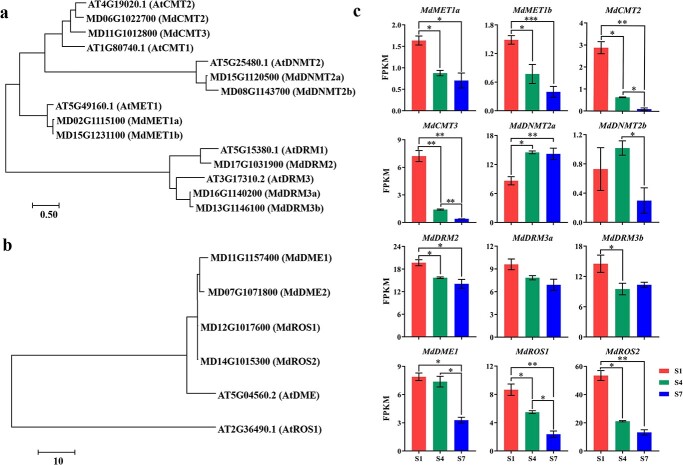
Analysis of DNA methyltransferase and demethylase genes. The phylogenetic trees were constructed using protein sequences of DNA methyltransferase (**a**) and demethylase (**b**). **c** Transcript levels of DNA methyltransferase and demethylase genes at three stages based on transcriptome analysis. Error bars are standard deviations of three biological replicates. ^*^P < 0.05; ^**^P < 0.01; ^***^P < 0.001.

### DNA methylation inhibitor treatment affects gene expression in flavonoid biosynthesis

To further confirm that changing DNA methylation can alter expression levels of genes in the flavonoid pathway, mature DH fruits were treated with the methylation inhibitor 5-aza-2′-deoxycytidine (5-aza-2dC) (Fig. 8a). Compared with water treatment (control), 5-aza-2dC treatment significantly increased anthocyanin levels and a* values of fruit from 6 days after the treatment (Fig. 8b and c). To evaluate DNA methylation levels of fruit treated with water or 5-aza-2dC, the *MdMYB10* promoter was selected to conduct an McrBC–PCR analysis. For the water-treated fruit, the PCR band signal for the MR3 region (−1250 to −784 bp) of the *MdMYB10* promoter was clearly reduced by McrBC plus GTP digestion compared with McrBC minus GTP digestion (Fig. 8d, left panel). McrBC can digest methylated DNA in the presence of GTP but cannot digest it without GTP. This result indicated that the methylation level of the MR3 region was relatively high. For the fruit treated with 5-aza-2dC, the PCR band signal showed no clear difference between the McrBC plus GTP and McrBC minus GTP treatment (Fig. 8d, right panel), indicating the 5-aza-2dC treatment inhibited DNA methylation. However, the methylation level of the other six regions (MR1, MR2, and MR4–MR7) of the *MdMYB10* promoter could not be clearly detected by McrBC–PCR analysis (Supplementary Data Fig. S7). We further divided MR3 into MR3C1 (−1012 to −737 bp) and MR3C2 (−1282 to −959 bp) to conduct bisulfite-PCR (BS-PCR) to quantify the methylation level (Fig. 8e and f). The methylation level was significantly reduced by treatment with 5-aza-2dC in the MR3C1 region (Fig. 8e) but not in the MR3C2 region (Fig. 8f). The expression level of genes involved in flavonoid accumulation, such as *MdCHS*, *MdCHI*, *MdANS*, *MdF3H*, *MdUFGT*, *MdMYB10*, *MdMATE*, *MdGST*, and *MdABC*, was significantly increased by 5-aza-2dC treatment, especially at 12 days after treatment (Fig. 8g).

### Increased DNA methylation may be caused by reduced expression of DNA demethylase genes

The relative levels of DNA methyltransferases and demethylases are critical factors in determining the plant DNA methylation level. We observed a general growth in DNA methylation levels during DH fruit development (Figs 6 and 7). To understand the underlying mechanisms, we examined the expression profiles of genes encoding these enzymes.

Using protein sequence BLAST analysis, we identified nine methyltransferases (MdMET1a/b, MdCMT2/3, MdDNMT2a/b, MdDRM2, and MdDRM3a/b) and four DNA demethylases (MdDME1/2, MdROS1/2) encoded in the apple genome. These proteins showed close relationships to six *Arabidopsis* methyltransferases (AtMET1, AtCMT1, AtCMT2, AtDNMT2, AtDRM1, and AtDRM3) (Fig. 9a) and two *Arabidopsis* DNA demethylases (AtDME and AtROS1) (Fig. 9b).

Transcriptome analysis uncovered that the transcript levels of *MdDNMT2b* were consistently low [<1 FPKM (fragments per kilobase of transcript per million mapped fragments)] across all three stages (S1, S4, S7). The transcript level of *MdDNMT2a* increased from S1 to S4/7, while the other seven methyltransferase genes showed decreased transcript levels during fruit development (Fig. 9c). *MdDME2* had a very low transcript level at S1 and was undetectable at S4 and S7. The remaining three demethylase genes, *MdDME1*, *MdROS1*, and *MdROS2*, exhibited significantly reduced transcript levels throughout fruit development (Fig. 9c). The expression profiles of these methyltransferase and demethylase genes generated by RNA-seq were confirmed by qRT–PCR (Supplementary Data Fig. S1).

Considering the positive correlation between DNA methyltransferase transcript levels and methylation levels, and the negative correlation between demethylase transcript levels and methylation levels, our findings suggest that the decreased expression of demethylase genes may contribute to the observed hypermethylation of the whole genome and promoters of genes in anthocyanin and PA accumulation during DH fruit development.

## Discussion

### Changes in gene expression profiles correlated with fruit color fading during DH fruit development

Fruit color fading is a fascinating phenomenon observed in horticultural products, and understanding the underlying mechanisms is of great interest. In our study, we investigated anthocyanin accumulation in the DH apple cultivar and found a gradual decrease in anthocyanin levels during fruit development. We also observed consistent declines in other flavonoids, such as chalcone (mainly phlorizin), catechin, epicatechin, naringenin-7-*O*-glucoside, procyanidin B2, and quercitrin. Interestingly, it was found in crabapple ‘Flame’ that the contents of phlorizin and procyanidin B2 decreased during fruit skin development, while epicatechin levels increased [42].

To uncover the molecular mechanisms underlying these changes, we performed transcriptome analysis and detected that the expression levels of most genes in anthocyanin biosynthesis decreased with fruit development. This observation correlated well with the changes in the levels of chalcone, naringenin-7-*O*-glucoside, quercitrin, and anthocyanin accumulation. Specifically, the reduced expression of *MdLAR*, *MdANR*, and *MdMYBPA1* corresponded to the decreased contents of catechin, epicatechin, and procyanidin B2 from the early (S1) to the mature stage (S7).

Apart from the decline in biosynthesis, the transport and degradation of anthocyanins also play a crucial role in final pigment accumulation [27]. In our study, we observed the repressed expression of transport genes *MdGST* and *MdABC* along with enhanced expression of degradation genes *MdLAC*, *MdPOD*, and *MdPPO*, which likely contributed to the color fading of DH apples. In the study of ‘Red Bartlett’ pears, increase in the expression of *LAC* and *POD* genes was reported during the color fading process, but no significant change was observed in *PPO* gene expression [40]. As PPO is primarily located in plastids, while anthocyanin degradation occurs in vacuoles, it is plausible that *MdLAC* and *MdPOD* were the main contributors to anthocyanin degradation in apple. Our findings highlight the dynamic changes in the expression profiles of genes in biosynthesis, transport, and degradation of anthocyanin during fruit development, shedding light on the mechanisms of fruit color fading.

### Role of MYB transcription factors in fruit color fading during DH fruit development

The regulation of flavonoid biosynthesis, particularly anthocyanin production, is predominantly governed by MYB transcription factors. Particularly, the expression levels of *MdMYB1/10* have been reported to positively correlate with anthocyanin content in apple skin and flesh [12–14, 18]. In the case of DH, promoter analysis identified *MdMYB1-2* and *MdMYB10*. However, it has been reported that *MdMYB1-2* cannot confer red skin color [43]. This could be attributed to the absence of the Red-TE insertion, which is the factor causing red fruit skin color in apples [18]. The absence of the Red-TE insertion has also been observed in red-fleshed apple genotypes (R6R6), in which red skin results from MdMYB10 autoregulation via binding to the R6 repeats in the promoter area [18]. Interestingly, in our study, only *MdMYB10* was detected from the cloned coding sequences of *MdMYB1/10*, while *MdMYB1-2* was not transcribed. Based on these findings, we deduced that the red skin color observed in R1R6 DH apples was primarily attributable to the expression of *MdMYB10*.

However, the expression of *MdMYB10* was not reduced together with fruit color fading during fruit development. A similar phenomenon was reported in red-fleshed apple (R1R6) previously. For instance, ‘Red Crisp 1’ has a significantly lower level of flesh coloration and anthocyanin compared with ‘Red Crisp 3’ and ‘Red Crisp 4’, although it has a higher level of *MdMYB10* expression. The inconsistency was due to the upregulation of the repressor *MdMYB16* [44]. Furthermore, it was found *MdMYB16*, *MdMYB17*, and *MdMYB111* could inhibit the autoregulation of *MdMYB10* [45]. Our study detected the upregulation of *MdMYB6*, *MdMYB16*, *MdMYB17*, *MdMYB28*, *MdMYB111*, and *MdMYB306-like* as fruit development progressed (Fig. 5). All these six MYBs were reported as transcription repressors [46–48]. It is most likely that the expression of the anthocyanin activator *MdMYB10* cannot fully compensate for the inhibiting role of the repressors with increasing expression, thus resulting in the observed color fading during fruit development.

Moreover, we observed that the highest transcription of *MdMYB1R1-like* was at S1 stage, which might also be responsible for the dark-red skin color. This finding aligns with studies conducted in ‘Red Delicious’ and its mutants, which also demonstrated a positive correlation between *MdMYB1R1-like* expression and anthocyanin accumulation [49]. Additionally, since MdMYBPA1 has been shown to regulate the promoters of not only *MdLAR* and *MdANR* but also *MdANS* and *MdUFGT* through interaction with MdbHLH33 [23], the high expression level of *MdMYBPA1* in S1 could potentially play a role in regulating the content of PAs and anthocyanins.

### DNA methylation is crucial in manipulating gene expression and anthocyanin biosynthesis

DNA methylation as a crucial epigenetic mechanism plays a significant role in plant growth and development, as well as fruit ripening and coloration [31–33, 50]. Generally, DNA methylation is connected with transcriptional silence, while the loss of DNA methylation can activate gene expression [51]. In our study we investigated the methylation levels of key gene promoters in anthocyanin and PA biosynthesis and found that the majority of these genes displayed a consistent pattern with the established regulatory relationship between promoter methylation and gene expression. For instance, promoters of structural genes such as *MdCHS*, *MdCHI*, *MdANS*, *MdF3H*, and *MdUFGT* showed increased methylation levels during fruit development, while their transcript levels were decreased. This finding is consistent with previous reports that show a negative relationship between transcript levels and promoter methylation levels of structural genes in peach fruits following storage at 16°C [52]. The treatment of mature DH fruit with 5-aza-2dC verified that the reduced DNA methylation increases the expression of *MdCHS*, *MdCHI*, *MdANS*, *MdF3H*, *MdUFGT*, *MdMYB10*, *MdMATE*, *MdGST*, and *MdABC* and further enhances anthocyanin accumulation.

Correlation analysis uncovered that the methylation of the *MdCHS* and *MdCHI* promoters not only negatively correlated with transcript levels but also with the corresponding chalcone and naringenin-7-glucoside content. This result is consistent with the previous finding that the methylation of the *MdCHS* promoter is negatively associated with gene expression and anthocyanin content in ‘Red Delicious’ and its mutants [49]. Regarding the promoter methylation of genes encoding MYB transcription factors, we observed a negative correlation between promoter methylation and gene expression levels in *MdMYBPA1* and *MdMYB1R1-like*. In addition, the use of ‘Red Delicious’ and its derivative four-generation mutants revealed that the CG methylation level of the novel MYB transcription factors *MdPCL-like* and *MdLUX* was negatively associated with their expression levels and anthocyanin content [53]. Overall, our findings support the notion that DNA methylation plays a vital role in manipulating gene expression and anthocyanin biosynthesis. The observed correlations between promoter methylation levels, gene expression, and anthocyanin content provide further evidence of the regulatory role of DNA methylation in fruit ripening and coloration processes.

### Changing expression profile of DNA methyltransferase and demethylase genes with fruit development

Dynamic DNA methylation in plants is regulated by the interplay between DNA methylation and DNA demethylation processes [54]. Our study showed a significant decrease in DNA demethylase gene expression during DH fruit development (Fig. 9). This decrease in DNA demethylase gene expression was consistent with the increased DNA methylation levels at a genome-wide scale and in the promoters of most of the examined genes. However, DNA methyltransferase genes were not abundantly expressed and did not show a strong correlation with the observed increased methylation levels. Similarly, reduction in transcripts of DNA demethylase genes was proposed as a potential mechanism for increased DNA methylation during sweet orange fruit ripening [32]. In contrast, upregulation of DNA demethylase genes was found to be a necessary requirement for tomato fruit development and ripening, through demethylation of critical gene promoters [33, 34]. In pepper, DNA hypomethylation of ripening-related gene promoters was observed at the turning stage, which was attributed to the upregulation of DNA demethylase genes and downregulation of DNA methyltransferase genes [55]. In strawberry, decreased methylation with fruit ripening was reported, and decreased siRNA levels and RNA-directed DNA methylation activity were proposed as the mechanisms underlying the observed demethylation [31].

The equilibrium between DNA methylation and demethylation processes, influenced by the expression levels of relevant genes, plays a crucial role in determining DNA methylation patterns and subsequent gene expression changes. The observed variations in DNA methylation dynamics among different plant species and even within different stages of fruit ripening emphasize the need for further investigations to fully understand the underlying regulatory mechanisms. Studying the interplay between DNA methylation and demethylation processes in different fruit crops can provide valuable insights for manipulating fruit quality attributes and improving agricultural practices.

## Materials and methods

### Plant materials

The plant material used in this research was a red-fleshed apple variety named DH that is selected from the hybrids of ‘Guifei’ (R1R6) and ‘Orin’ (R1R1). The plants were 8 years old and grown on the *Malus robusta* rootstock at the Jiaozhou agricultural demonstration station of Qingdao Agricultural University. Fruits of DH were collected at seven different stages: 30 (S1), 45 (S2), 55 (S3), 70 (S4), 90 (S5), 110 (S6), and 130 (S7) days after full bloom (DAFB). Each stage had three biological replicates. The fruit skin was sampled, then immediately placed in liquid nitrogen and stored at −80°C.

### Extraction and determination of anthocyanins

Anthocyanins were extracted from the fruit skin by incubating 0.5 g of tissue with 1% (v/v) methanol–HCl with ratio 1:10 (w/v) at 4°C under darkness for 15 h. The extracted anthocyanins were then filtered (0.45-μm membrane) and stored at −4°C. For anthocyanin content examination, the pH differential method was applied, the detailed procedure being based on previous publications of our research group [56].

### Determination of flavonoid components by UPLC–MS/MS

The freeze-dried DH fruit peel was ground to powder, and 20 mg of powder from each sample was used. A total of 500 μl of methanol (70%) was added to the powder and the sample was thoroughly mixed. The samples were then subjected to ultrasonication for 30 min, then centrifuged for 5 min at 15 456 rcf. The supernatant was collected and transferred into vials after filtration using a 0.22-μm pore size filter (Sangon Biotech, Shanghai, China).

The compounds were analyzed using an ExionLC analytical UPLC system combined with a Qtrap 6500 mass spectrometer (AB Sciex, CA, USA). The analytical conditions used were (i) a Waters ACQUITY UPLC BEH T3 C18 column (Waters Corporation, MA, USA); (ii) solvent systems A (0.05% formic acid) and B (0.05% formic acid in acetonitrile); (iii) gradient program: A/B (90:10 v/v) at 0 min, 80:20 v/v at 1 min, 30:70 v/v at 9 min, 5:95 v/v at 12.5 min, 5:95 v/v at 13.5 min, 90:10 v/v at 13.6 min, 90:10 v/v at 15 min; (iv) flow rate 0.35 ml/min, temperature 40°C, injection volume 2 μl.

The quantification of flavonoid component contents was according to the peak area as well as a linear equation derived from standard curves. The calculation was as follows: content of component (mg/kg) = *c* × *V*/*m*, where *c* represents the concentration of the analyzed component, *V* denotes the solution volume (μl), and *m* represents the weight of the sample (μg).

### DNA extraction and whole-genome bisulfite sequencing

Genomic DNA was obtained from DH fruit skin samples collected at three stages (S1, S4, and S7) using the cetyltrimethyl ammonium bromide (CTAB) method with modifications [57]. The extracted DNA was subjected to EZ DNA Methylation-Gold Kit (Zymo Research) treatment before preparation of libraries. The prepared libraries of DNA samples were sequenced on the Illumina Novaseq platform by OE Biotech Co., Ltd (Shanghai, China). The obtained reads were aligned to the apple reference genome using Bismark software (version 0.16.3) [58]. The weighted methylation level was calculated based on a previously reported method [59].

### Analysis of *MdMYB1/10* promoter and coding sequences

The promoter of *MdMYB1/10* was amplified from leaf DNA using FP 5′-GGAGCTCACTAGCTTCGGATTCCT-3′ and RP 5′-GGTTTTCGTTATATCCCTCCATCT-3′. The cDNA of *MdMYB1/10* was amplified from fruit skin cDNA using FP 5′-CAGATAAGAGATGGAGGGATAT-3′ and RP 5′-ATCCCACATTTACAAGCAAGG-3′. After electrophoresis, the PCR products of promoter and cDNA of *MdMYB1/10* were purified, then constructed into cloning vector pMD 18-T (TaKaRa) and sequenced.

To detect Red-TE insertion in the *MdMYB1/10* promoter, genomic DNA of apple cultivars was analyzed by PCR using two forward primers and one reverse primer as previously described [60]. The F1 primer (5′- CGGATTGTTCCTGCTGTCTCTCTGTTGACA-3′) was located in Red-TE region and could amplify a fragment of 386 bp with R1 primer (TTTTCCCTTCATTGAGCACTAATTTTC). The F2 primer (5′-ATATCACACTCCCTTCTCTTTCTAG-3′) was not in the Red-TE region and could amplify a fragment of 250 bp with R1 primer.

### Transcriptome analysis

Total RNA was extracted from DH fruit skin collected at three different stages (S1, S4, and S7) using the RNAprep Pure Plant Kit (DP441, Tiangen Biotech). The extracted RNA was quantified using Qubit RNA BR Assay Kits and a Qubit 2.0 Fluorometer (Invitrogen, Eugene, OR, USA). The integrity of the RNA samples was assessed using the RNA 6000 Nano labchip on the 2100 Agilent Bioanalyzer (Agilent Technologies, CA, USA). Nine RNA-seq libraries, representing the three stages and their biological replicates, were prepared using the TruSeq Stranded mRNA LT Sample Prep Kit (Illumina, CA, USA) then sequenced using the Illumina HiSeq X Ten platform.

Trimmomatic version 0.32 was used to filter and trim raw sequencing reads. The obtained high-quality clean reads were then aligned to the apple GDDH13 reference genome using HISAT2 [61]. HTSeq was used for calculation of the read counts mapped to genes, and the quantification of expression levels was according to FPKM using Cufflinks [62]. Differential expression analysis was performed using the R package Bioconductor DESeq2, with a cutoff of adjusted *P*-value (FDR) < 0.05 plus |log_2_FoldChange| > 1 for identifying DEGs [63].

### DNA methylation inhibitor treatment, McrBC–PCR, and BS–PCR analysis

DH fruits at the mature stage were collected and placed in darkness for 24 h in a chamber before treatment. The fruit surfaces were treated with 1 mM 5-aza-2dC (Sigma–Aldrich, USA) and 0.1% (v/v) Tween-20 or just Tween-20 as the control group. The conditions of the chamber were set as: light (250 μmol m^−2^ s^−1^) for 16 h at 16°C, then darkness for 8 h at 25°C. Apple skin color was measured by using a colorimeter (Konica Minolta CR-400), producing a* values that represent red when positive (+) and green when negative (−). The fruit skin was sampled at 0, 6, 12, and 16 days after the 5-aza-2dC treatment, with three biological replicates and four fruits for each replicate.

Genomic DNA was extracted as previously described. For McrBC–PCR, the DNA was digested by using the methylation-dependent enzyme McrBC (New England Biolabs) in the presence or absence of GTP and then used as template for PCR. GTP is required for McrBC activity. The PCR primer pairs were designed to amplify seven specific regions (MR1–MR7) of the *MdMYB10* promoter (Supplementary Data Table S4). The DNA methylation level was evaluated according to the relative strength of the PCR band signal between McrBC digested with GTP and McrBC digested without GTP.

For BS–PCR, DNA was treated with the EZ DNA Methylation-Gold Kit (Zymo Research) to convert unmethylated cytosine to uracil, and unmethylated lambda DNA was spiked as reference for conversion efficiency. BS–PCR (50 μl) was conducted with converted DNA and degenerate primers, which are listed in Supplementary Data Table S5. The PCR products were cloned into the vector pMD18-T after purification, and then sequenced. The cloned sequences were aligned to the reference sequence. CyMATE was used for visualization and calculation of the methylation patterns and levels [64].

### Quantitative real-time PCR validation

A total of 1.5 μg RNA was used for first-strand cDNA synthesis using 5X All-In-One RT MasterMix (abm, China). Gene-specific primers and ChamQ SYBR Color qPCR Master Mix (Vazyme, Shanghai, China) were used to conduct qRT–PCR (Supplementary Data Table S6). The qRT–PCR reactions of three independent biological and three technical replicates were performed using a CFX96 instrument (Bio-Rad, CA, USA). The data were analyzed based on the 2^−ΔΔCt^ method, which allowed the calculation of relative gene expression levels normalized to the reference gene.

### Statistical analysis

The data are displayed as mean ± standard error. Correlation analysis between DNA methylation of promoters and corresponding transcript levels, as well as DNA methylation of promoters and metabolite content, was conducted using Pearson’s correlation coefficient. The statistical analysis was conducted using SPSS 21.0 software (Chicago, IL, USA).

## Acknowledgements

This work was supported by the Natural Science Foundation of Shandong Province (ZR2023MC169), the Taishan Scholar Foundation of Shandong Province (tstp20221134), the Agricultural Variety Improvement Project of Shandong Province (2023LZGCQY007; 2021LZGC024; 2022LZGC010), the China Agriculture Research System Foundation (CARS-27), and Science and Technology Specific Projects in Agricultural High-tech Industrial Demonstration Area of the Yellow River Delta (2022SZX34).

## Author contributions

J.X. wrote the manuscript. J.L.Y. revised the manuscript. J.X., Y.Z., and J.L.Y. designed the experiments. L.X., P.Z., J.X., S.J., X.S., C.D., H.J., and X.X. performed the experiments and analyzed the data. All the authors approved the final manuscript.

## Data availability

The transcriptome and methylome data in this study have been deposited in the Sequence Read Archive (SRA) database in NCBI, with bioProject accession numbers PRJNA1000056 and PRJNA1000046, respectively.

## Conflict of interest

The authors declare that there is no conflict of interest associated with this work.

## Supplementary data

Supplementary data is available at *Horticulture Research* online.

## Supplementary Material

Web_Material_uhae031
