# Supplementary material for: Hypermethylation in the promoter regions of flavonoid pathway genes is associated with skin color fading during ‘Daihong’ apple fruit development
Source: Hortic Res. 2024 Feb 15;11(3):uhae031. doi: 10.1093/hr/uhae031 (PMC10933707; doi:10.1093/hr/uhae031)
Supplement: Web_Material_uhae031 [file web_material_uhae031.zip › supplementary figures legends.docx]

**Figure S1.** Validation of gene expression profile by comparing the results of RNA-Seq and qRT-PCR assay. Error bars are standard deviations of three biological replicates.

**Figure S2.** The epigenome profile of ‘DH’ fruit skin at different stages. Red, green, black and blue layers indicated profiles of mC, mCG, mCHG, mCHH across chromosome.

**Figure S3.** Hyper- and hypo-differentially methylated regions (DMRs) in CHH context in three comparative groups in circos plots and heatmap clusters.

**Figure S4.** KEGG pathway enrichment analysis of DMRs in three comparative groups.

**Figure S5.** Increased methylation levels of promoter region of key genes involved in flavonoid biosynthesis from S1 to S7 as viewed by IGV.

**Figure S6.** Analysis of DNA methylation levels in the MR3 region of *MdMYB10* promoter with McrBC-PCR. *MdMYB10* promoter was divided into seven regions (MR1-MR7). S1, S4 and S7 represent 30, 71 and 131 days after full bloom (DAFB). The DNA was digested by McrBC enzyme with GTP (M) or without GTP as a control (C). Reactions were conducted with two biological replicates.

**Figure S7.** Analysis of DNA methylation levels in the MR1, MR2, and MR4 to MR7 regions of *MdMYB10* promoter after 5-aza-2-dC treatment by using McrBC-PCR assay. The DNA was digested by McrBC enzyme with GTP (M) or without GTP as a control (C). Reactions were conducted with three biological replicates.
