# Supplementary figures and images for: Hypermethylation in the promoter regions of flavonoid pathway genes is associated with skin color fading during ‘Daihong’ apple fruit development
Source: Hortic Res. 2024 Feb 15;11(3):uhae031. doi: 10.1093/hr/uhae031 (PMC10933707; doi:10.1093/hr/uhae031)

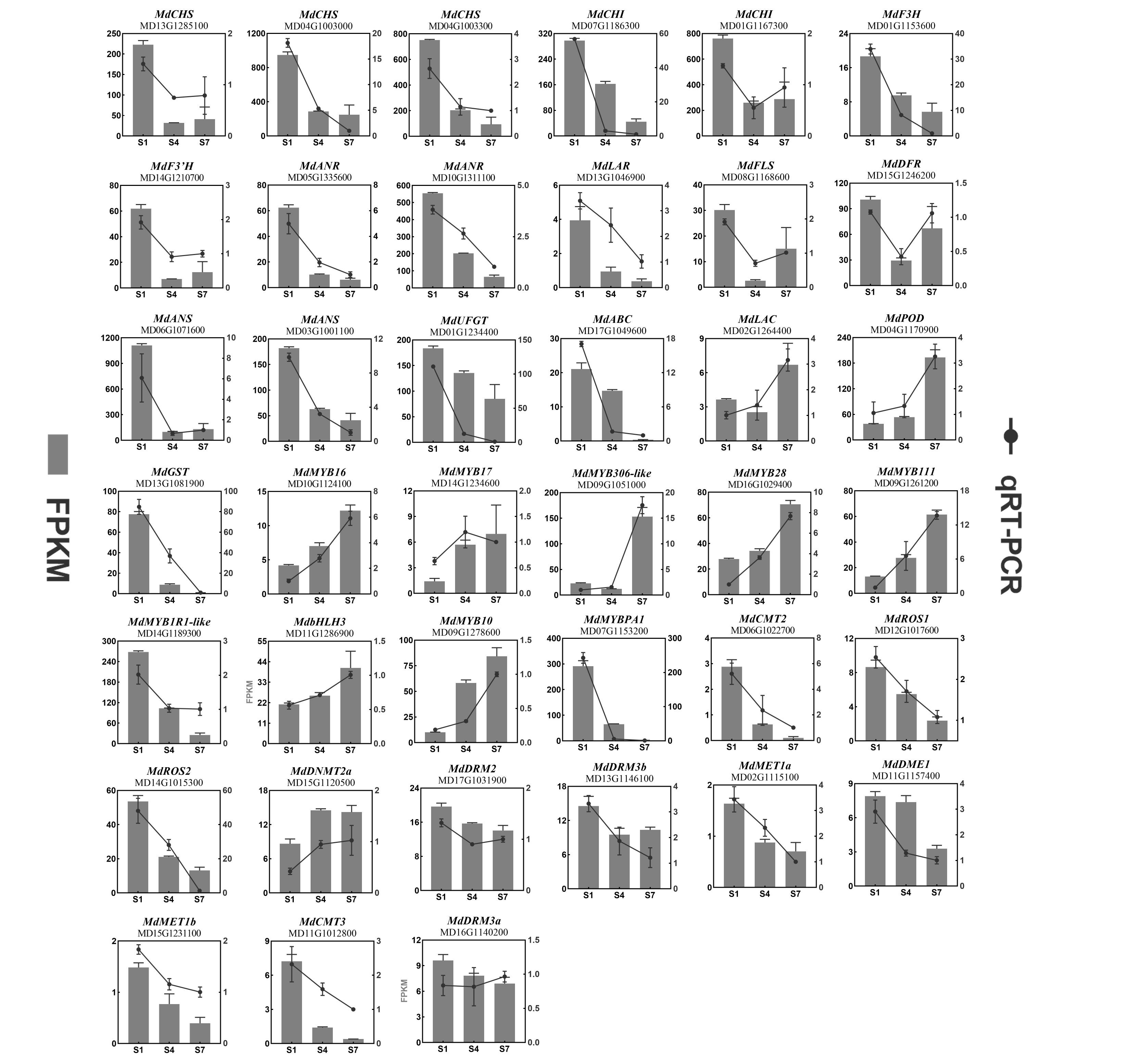

Supplement: Web_Material_uhae031 [file web_material_uhae031.zip › Figure S1.jpg]

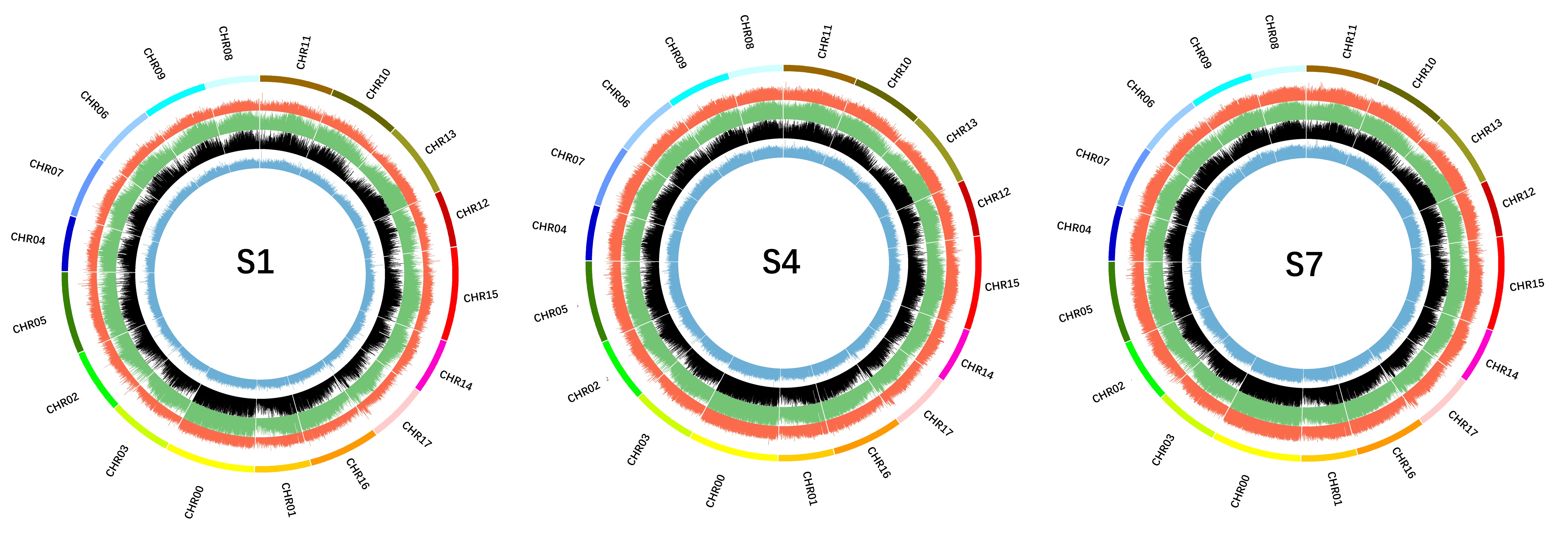

Supplement: Web_Material_uhae031 [file web_material_uhae031.zip › Figure S2.jpg]

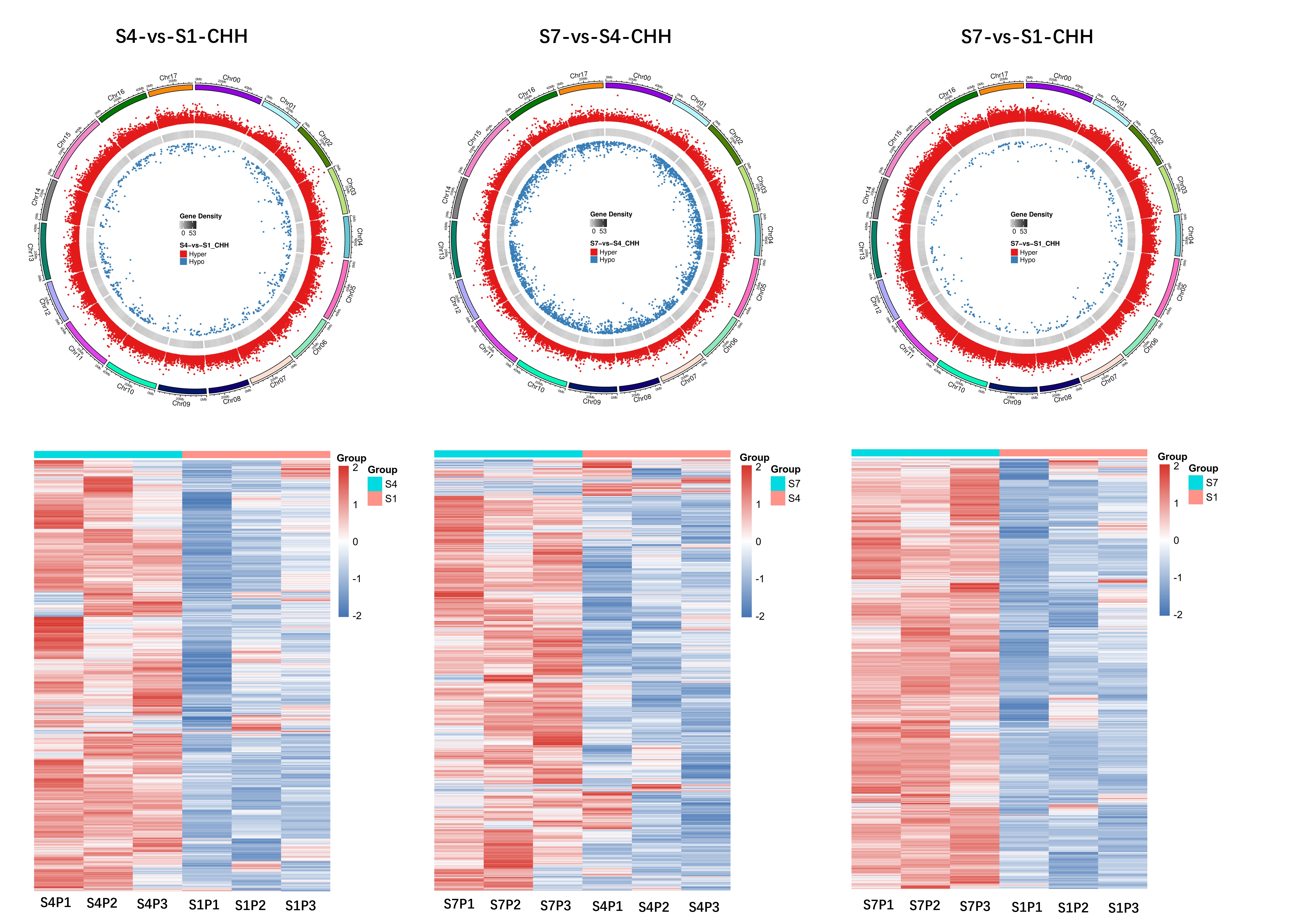

Supplement: Web_Material_uhae031 [file web_material_uhae031.zip › Figure S3.jpg]

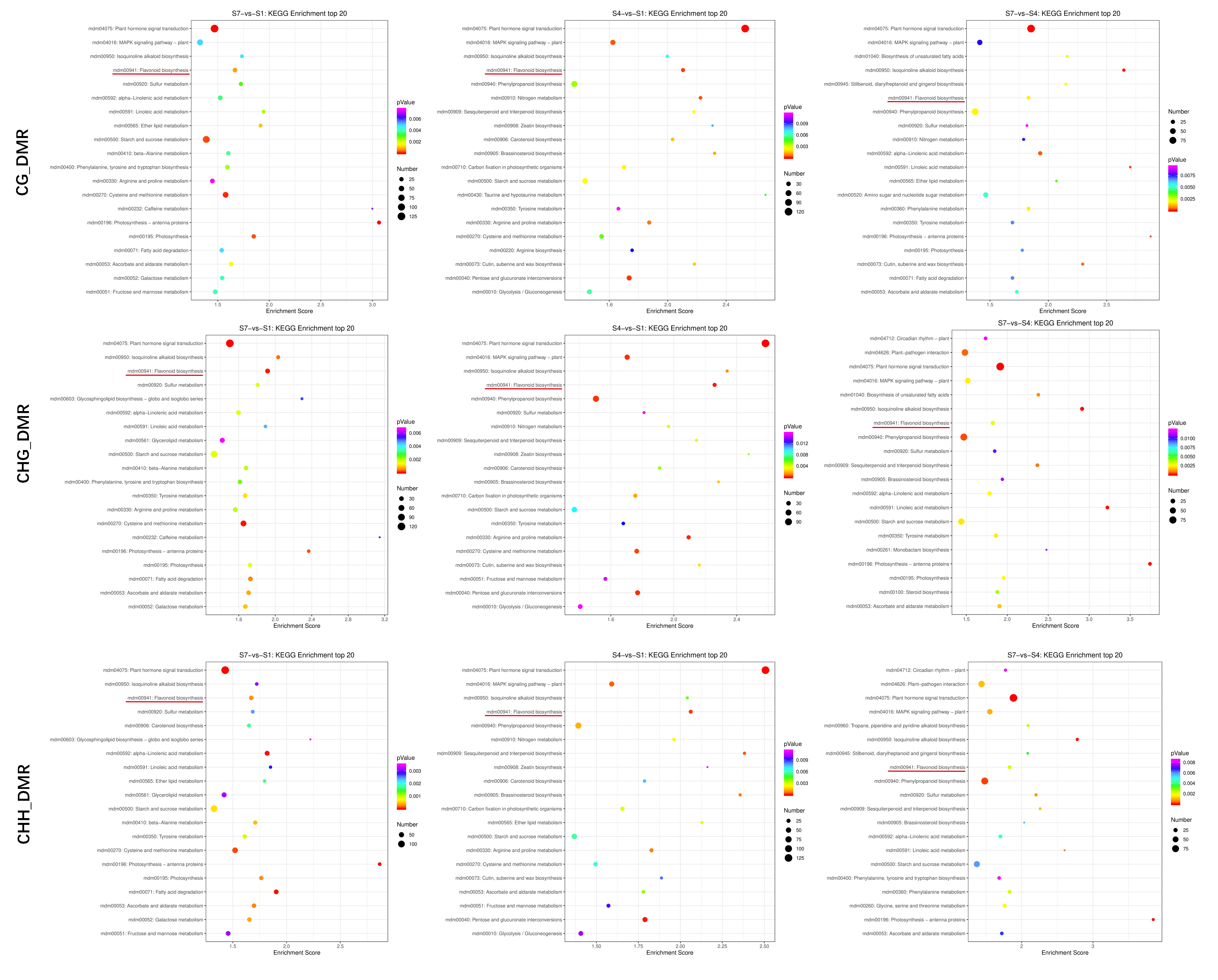

Supplement: Web_Material_uhae031 [file web_material_uhae031.zip › Figure S4.png]

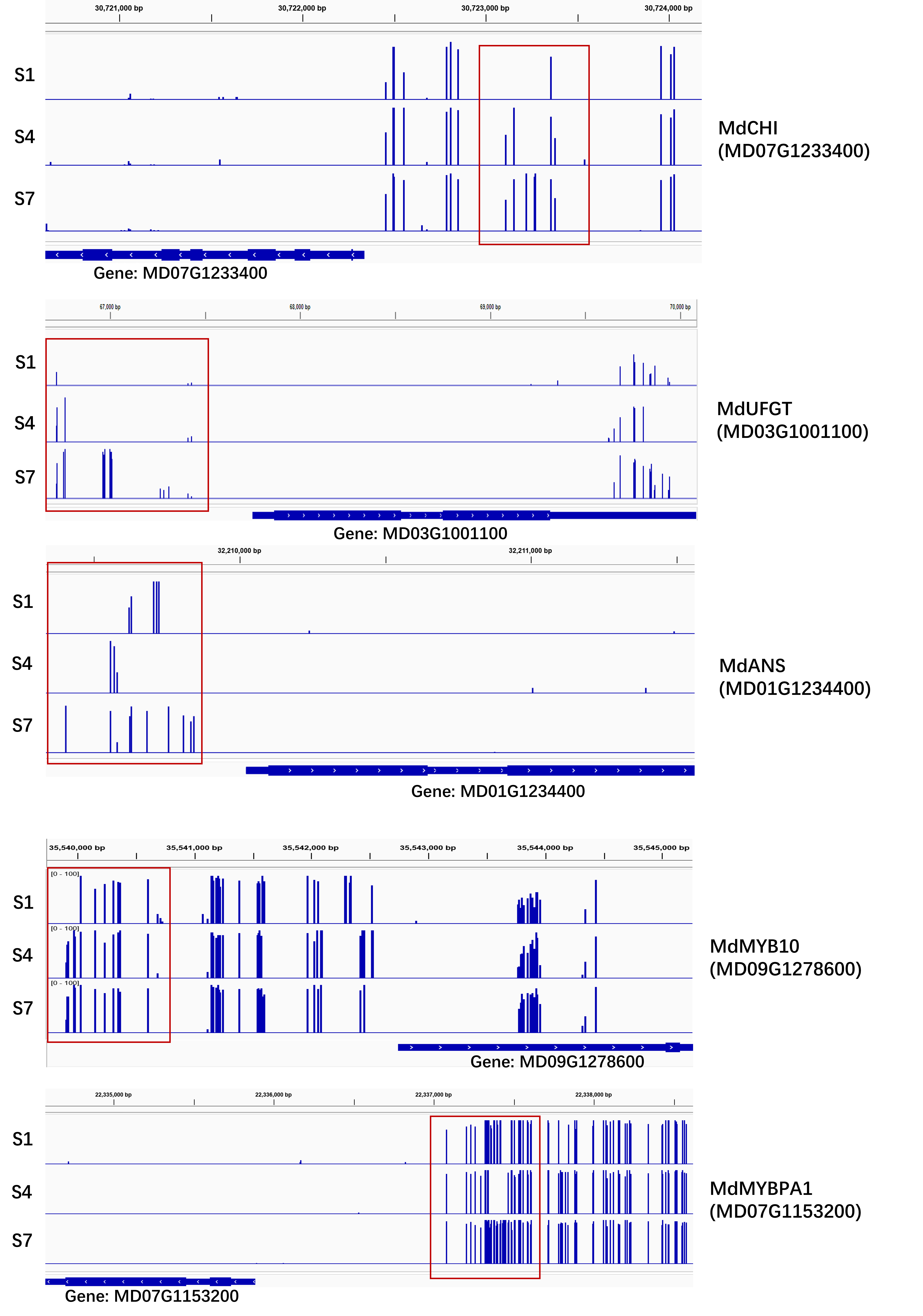

Supplement: Web_Material_uhae031 [file web_material_uhae031.zip › Figure S5.png]

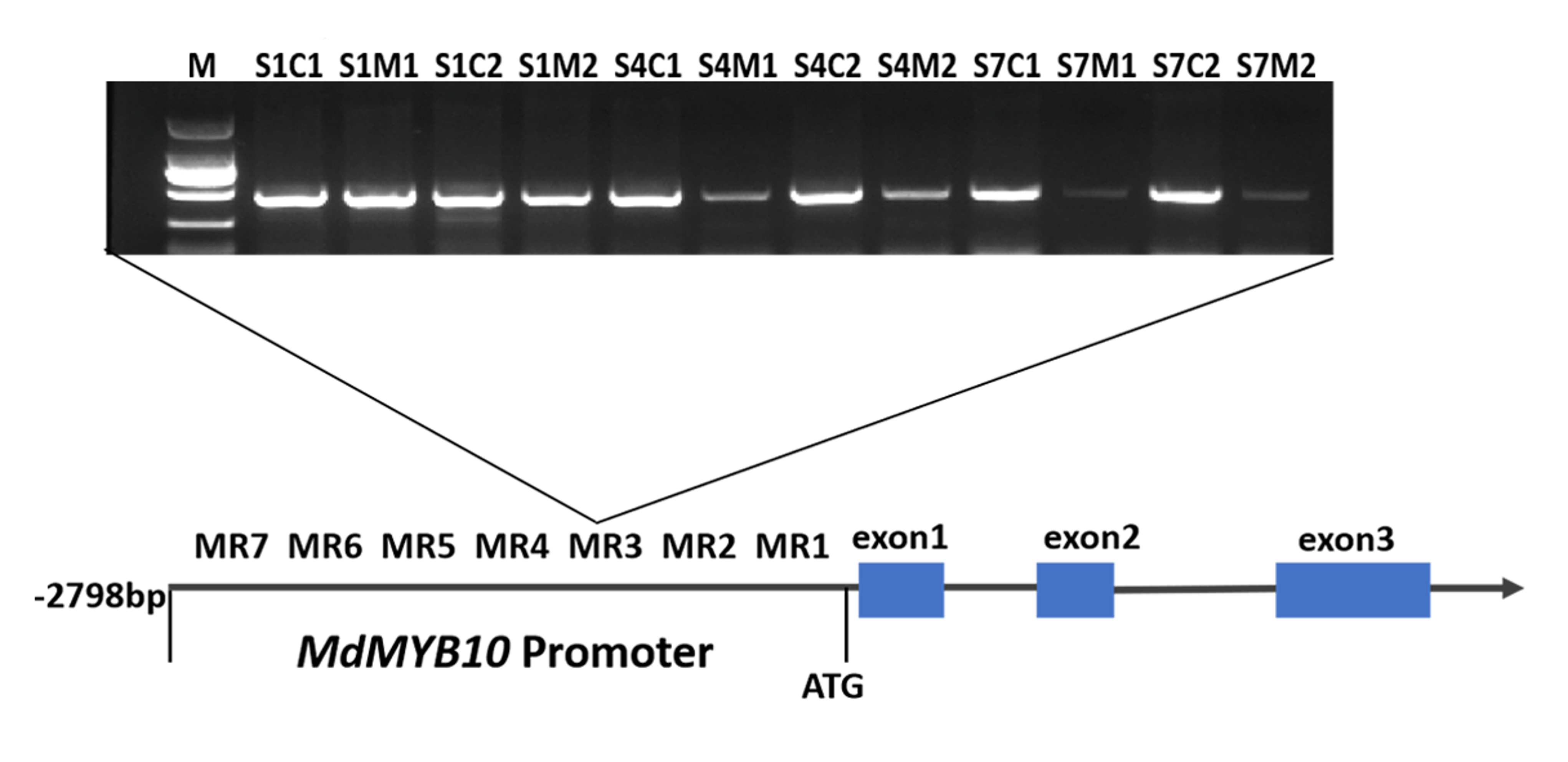

Supplement: Web_Material_uhae031 [file web_material_uhae031.zip › Figure S6.png]

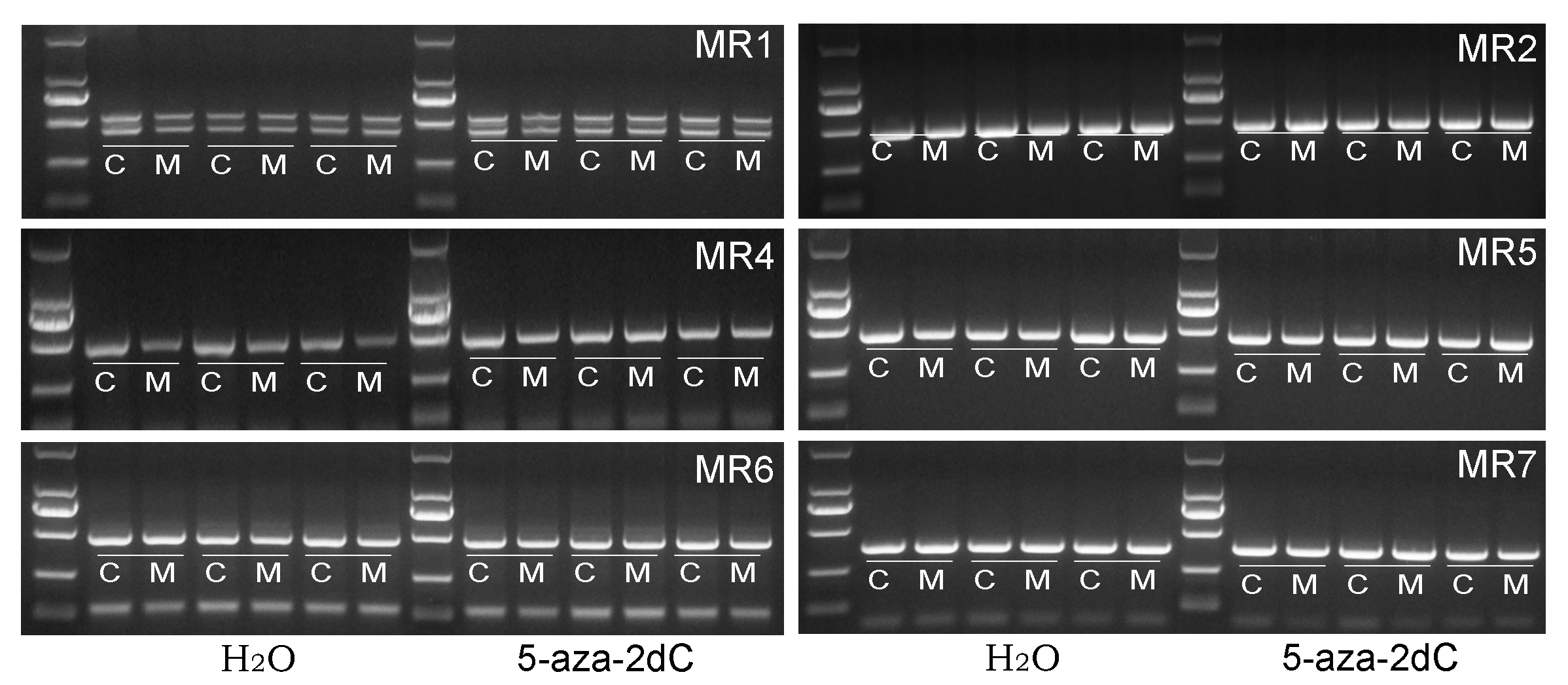

Supplement: Web_Material_uhae031 [file web_material_uhae031.zip › Figure S7.png]
